# Sarcomatoid Carcinoma of the Penis: An Uncommon Penile Neoplasm

**DOI:** 10.30699/ijp.2020.117401.2275

**Published:** 2020-02-28

**Authors:** Sucheta Gandhe, Rahul Patil, Raj Nagarkar

**Affiliations:** 1 *Department of Pathology, HCG Manavata Cancer Centre, Nashik, Maharashtra, India*; 2 *Department of Surgical Oncology, HCG Manavata Cancer Centre, Nashik, Maharashtra, India*

**Keywords:** Immunohistochemistry, Penis, Sarcomatoid carcinoma

## Abstract

Sarcomatoid squamous cell carcinomas are extremely rare, high grade, aggressive variant of penile cancers. Sarcomatoid carcinoma are biphasic neoplasms with a combination of both sarcomatoid components and carcinomatous elements. These neoplasms are very rare in the urogenital system. We report a 53-year-old male presented with an ulcerated lesion on the glans penis. The rarity of this case reiterates the importance of thorough morphological and histological examination along with immunohistochemistry in diagnosing, staging, treatment and follow up of patients.

## Introduction

Penile cancer is a rare neoplasm with an annual incidence varying from 0.2 to 1 per 1,00,000 men, worldwide annually (1). Among all penile cancers, 95% of the cases histologically correspond to squamous cell carcinomas (SCCs). Remaining 5% are sarcomatoid carcinomas (SC), which are extremely aggressive and rare form of penile cancers. SC patient's age widely ranges from 28 to 83 years and the glans penis is the most frequently affected area. Only 40 cases of sarcomatoid penile carcinoma have been reported worldwide to date (2,3). 

SC has also been called metaplastic, spindle cell, or biphasic SCC. Most pathologists today accept that SC as a tumor, originates from an epithelial cell. They also express both mesenchymal and epithelial antigens when tested by immunohistochemistry (IHC) (4,5). 

Here we are reporting a patient with penile SC treated at our institute (HCG Manavata cancer centre, India) with the necessary clinicopathologic correlations. It’s a clinically significant case because of its aggressiveness, rarity, clinicopathologic curiosity and lower survival rate.

## Case Report

A 53-year-old male was presented to our hospital with an ulcerated lesion on the glans penis over past 3 months. A 4 × 3.5 cm ulcerated neoplasm involving the dorsal aspect of the glans penis was revealed on clinical examination. The urethral meatus and penile shaft are free from neoplasms. There were significantly enlarged nodes approximately measuring 4 × 4 cm in the left inguinal region. 

A CT revealed heterogeneously enhancing lesion measuring 3.9 × 3.7 × 4.1 cm in glans penis contiguous distal end of corpora cavernosa and spongiosum. Multiple enlarged lymph nodes were observed in the bilateral external iliac and inguinal region with the largest measuring 3.2 × 3.1 cm in the left inguinal region. 

The patient underwent a partial amputation with groin node dissection in May 2018. The patient had an uneventful postoperative recovery. 

**Fig. 1:**
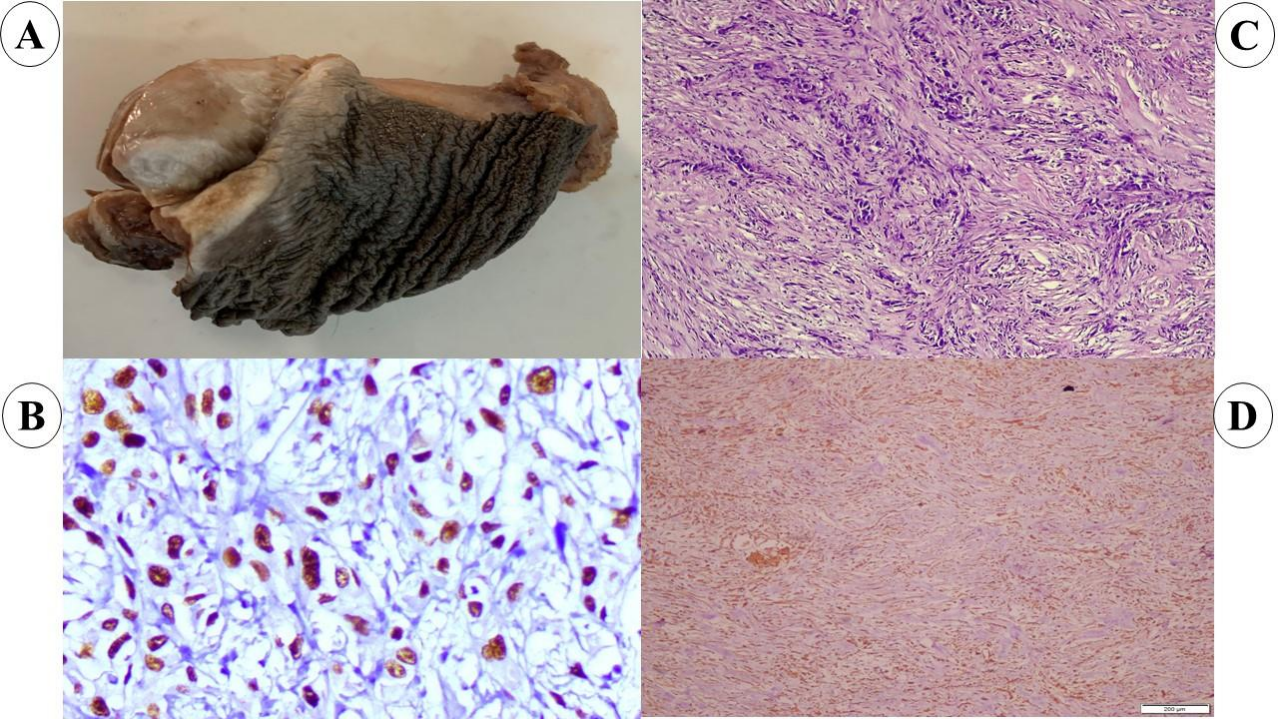
(A) Gross histopathological specimen of partial penectomy. Ulcerated lesion involving dorsal aspect of glans. (B) H and E (10x) show atypical squamous epithelial cells admixed with spindle cells. (C) IHC for P63 show strong nuclear positivity by malignant squamous epithelial cells. (D) Sarcomatous component of the tumor is highlighted by Vimentin

Gross features such as ulcero-proliferative growth was observed on the glans penis (Figure 1A). Histological hematoxylin and eosin (H&E) stained sections has shown features of a high-grade pleomorphic spindle cell neoplasm with high mitotic activity (15 to 20 mitosis/10 HPF).  The tumor cells were arranged in interlacing fascicles with moderate eosinophilic cytoplasm. Occasional areas of atypical squamous epithelial cells were observed (Figure 1B). Histopathologic (HP) reports have shown tumor as a poorly differentiated carcinoma with nodal involvement.

Immunohistochemistry (IHC) showed positivity for P63 (Figure 1C), P40 and Vimentin (Figure 1D). The tumor cells were negative for desmin, HMB45, and smooth muscle actin.

Based on the IHC and HP findings, a diagnosis of SC of the penis was made. 

The patient received CTRT 50 Gray in 25 fractions with concurrent cisplatin (5 cycles), post-operative till July 2018. After 10 months of regular follow-up, CT Pelvis was done which revealed bilateral external iliac and left para-aortic lymphadenopathy. The biopsy of the iliac node revealed metastatic SC in a known case. Bilateral retroperitoneal lymph node dissection was done in May 2019. The patient received an additional three cycles of gemcitabine and carboplatin.

Based on the initial diagnosis, the best possible treatment was selected and initiated as per clinical practice guidelines laid by the National Comprehensive Cancer Network (NCCN). The patient is currently doing well and due for follow-up.

## Discussion

 SC is an unusual, large and aggressive tumor associated with a higher rate of lymph node metastasis and poor prognosis (6). Lymph node or distant metastasis was reported in many cases, suggesting that marked vascular infiltration is a cause of the poor prognosis. Apart from direct hematogenous and lymphatic spread, “Satellitosis” is another mode of metastasis, which is commonly observed in patients with SC, and high-grade SCCs (7,8). 

The penile neoplasm morphological features are similar to their counterparts in other locations, with higher number of malignant spindle cells and lower number of squamous cell components as in our case. Sarcomatoid carcinoma are biphasic neoplasms with a combination of both sarcomatoid components and carcinomatous elements (1). As they have both epithelial and mesenchymal components, dissemination occurs via the hematogenous and lymphatic systems leading to both regional and distant metastasis (5). 

Positive prognostic factors can always be correlated with the overall survival of the patients. The patient's prognosis depends on various prognostic factors such as size, nuclear grade, nodal metastasis, tumor stage, and chemokine receptors expression (i.e., CXCR2 and CXCR3), etc. (1,3,5,9). Positive prognostic factors for tumorigenesis in SC of the penis can be correlated with similarities observed in other SC cases i.e., renal cell carcinoma (9). An accurate, thorough morphological, HP, and IHC examination is highly recommended in diagnosing, staging, treatment and follow-up of patients.

Risk factors for SC of penis include human papillomavirus (HPV) infection, poor hygiene, and phimosis. Intratumoral vascular proliferation, lymphovascular emboli, perineural invasion, and overexpression of chemokine receptors may cause metastasis (10).  Bijan *et al.* observe the clinicopathologic factors of patients and prognostic value of the expression of CXCR2 and CXCR3 markers in SC of renal cases. Results from their study have correlated and concluded that higher expression of CXCR3 and CXCR3 was observed with disease progression, which in turn lead to shorter overall survival (9).

In a retrospective study conducted by Lont *et al.* in 2004, the incidence of SC of the penis (1.4 %) was observed to be very rare (5 cases, over a span of 46 years), and they have studied all its related IHC, morphological, and clinical features over the years in all the reported cases (5). In another study, Velazquez *et al.* had defined and evaluated the clinicopathological features of 15 SC cases (4%) on a retrospective analysis of 400 cases of squamous cell carcinoma of the penis (3). Many patients were presented with either focal or distant metastasis, with a mortality rate of 50–80% (3,5). In the present case, our treatment protocol goes along with previously conducted studies. Overall our findings from the case are in agreement with the previously reported studies (1,2,3,5,8). Early diagnosis and treatment is the only coping method. Palliative surgery may be considered in patients with ulcerated advanced tumors, which will help them in providing temporary relief from pain, bleeding and tumor regression over a period of time (1).

## Conclusion

SC of the penis is an unusual entity while the mainstay of treatment remains surgery. SC of the penis is associated with poor prognosis. SC should be differentiated from poorly differentiated squamous carcinoma, melanoma, and sarcoma. An accurate, thorough morphological and histological examination along with IHC would help in diagnosing this rare aggressive entity.
